# Barriers and leverage points for seeing alcohol differently in integrated care systems in England: a senior stakeholder interview study

**DOI:** 10.1136/bmjph-2023-000829

**Published:** 2024-07-31

**Authors:** Mary Madden, Duncan Stewart, Jim McCambridge

**Affiliations:** 1 University of York, York, UK; 2 London Metropolitan University, London, UK

**Keywords:** Public Health, Primary Prevention, Secondary Prevention, Tertiary Prevention

## Abstract

**Introduction:**

Integrated care systems (ICSs) are the latest major innovation aiming to develop localised, integrated health and social care services to improve population health in England. Nationally, alcohol has received limited attention in National Health Service (NHS) strategic decision-making relative to its burden of harm, which varies considerably in localities. We examined decision-making and progress on alcohol in two contrasting ICSs, identifying systemic barriers to dealing with alcohol harm and potential leverage points, particularly in primary care.

**Methods:**

Qualitative case study in two ICSs differing in strategic prioritisation of alcohol in Northern England. In-depth semistructured interviews with 14 senior stakeholders followed by constructionist thematic analysis.

**Results:**

ICS formation occurred when services had been under sustained pressures with lines of communication and accountability emergent and unclear. Stakeholders identified fundamental disconnects between prevention and treatment. ICS strategic prioritisation of alcohol engendered new perspectives and novel actions. Even where not prioritised, there was a demand for placing alcohol work within a population frame. Attention to alcohol was somewhat precarious in primary care and overlooked in NHS health inequalities discourse. Reframing alcohol clinically as a drug was seen as having unrealised potential to prevent or delay disease onset and complications and improve NHS effectiveness. While congruent with the vision of how the new system should be working, there were doubts about capacity in current circumstances.

**Conclusions:**

There is much to do to create a joined-up, system-wide approach to alcohol, and thus a strong case for a national NHS alcohol strategy to guide ICS decision-making, addressing links between NHS work and public health.

**WHAT IS ALREADY KNOWN ON THIS TOPIC**

**WHAT THIS STUDY ADDS**

- There is widespread recognition of the need to place alcohol within a population frame to address its somewhat precarious position in primary care and being overlooked in policy discourses.
- It is possible to engender new perspectives and novel actions on alcohol within the ICS structure.
- There is much to do to create a joined-up, system-wide approach to alcohol, without which progress will be avoidably restricted.

**HOW THIS STUDY MIGHT AFFECT RESEARCH, PRACTICE OR POLICY**

- There is scope for ICSs to be important agents of change in addressing alcohol as a population-level issue, and national guidance has an important role to play in supporting this process.

## Introduction

 Since 1 July 2022, local authorities in England have been working in partnership with the National Health Service (NHS) in new integrated care systems (ICSs). The purpose of ICSs is to provide more joined-up action to improve the health and care of people in their area, with a focus on prevention, better outcomes and reducing health inequalities, involving local community and voluntary organisations.^1^ A 2023 review of progress identified prevention as important, but a poor relation in emerging ICSs, because of challenges in meeting basic clinical and social care needs in the wake of austerity policies and COVID-19.^2^ A recent rapid review of prevention identified commonly recurring programme level barriers, compounded by system level issues which include lack of integration, infrastructure and communication.^3^ The rhetorical commitment to prevention has not been matched with material investment.^2^ NHS funding remains overly focused on treatment of illness or injury rather than prevention, and ICS partners struggle with complex, uncoordinated funding systems and rules.^2^ Similarly, attention to the roles of primary and community healthcare has been criticised as not being followed by clear actions,^4^ and that underinvestment in prevention in primary care is short sighted.^5^ Rising demand, declining numbers of general practitioners (GPs) and recruitment and retention struggles in England are now well recognised.^6^

Approaches to alcohol policy vary considerably across the UK, and treatment systems are not conceptualised within a wider public health framework.^7^ For example, in England, there is no strategy to implement population measures, such as restricting pricing, availability and marketing as per the Global Alcohol Action Plan which the UK government signed in May 2022 at the 75th World Health Assembly.^7^ Combatting Drugs Partnerships (CDPs), introduced at the same time as ICSs in June 2022,^8^ focus on local, multiagency approaches to reducing the use and supply of illegal drugs. Alcohol is noted as another factor in many drug-related deaths, with CDP guidance suggesting that plans sufficiently address alcohol dependence and wider alcohol-related harms.^9^

Alcohol is an addictive, toxic and psychoactive drug. It is a major contributor to the global burden of ill health, including in long-term non-communicable diseases.^10 11^ Although heavy drinkers are most at risk, all drinkers are at risk of alcohol harms, as are non-drinkers around them. The NHS alcohol prevention agenda has been driven by the Long Term Plan (LTP) which notes that, “alcohol contributes to conditions including cardiovascular disease, cancer and liver disease, harm from accidents, violence and self-harm, and puts substantial pressure on the NHS.”^12^ Nevertheless, the LTP is focused narrowly downstream on alcohol dependence-related admissions to hospital.^12^ Alcohol screening and brief intervention programmes in primary care seem attractive for prevention but have been challenging to implement in routine practice.^13^

The recent NHS England Core20PLUS5 approach to reducing healthcare inequalities at national and system level mentions alcohol dependence, but not that alcohol is also directly implicated in the key clinical areas it identifies as requiring accelerated improvement: hypertension, mental illness, cancer, COPD and maternity services.^14^ ICSs are left to determine their own approaches to alcohol within a framework where alcohol continues to be something of a blind spot as a key health risk factor requiring long-term, genuinely strategic attention.^15 16^ Many staff asked to intervene on alcohol in the NHS have little substantial training, see public health as separate to their clinical role and have limited evidence-informed knowledge of the subject.^17^,^19^ This study explores views on roles and practices relating to alcohol within the new ICS infrastructure as part of a research programme tackling the management of alcohol in primary care.^20 21^

## Methods

With the support of North of England Commissioning Support Unit, we recruited and conducted interviews with 14 key stakeholders in senior leadership roles in two contrasting sites in Northern England. One area, that is, ICSa, had a relatively well-developed ICS and its integrated care board (ICB) had prioritised alcohol, while the other area, that is, ICSb, had no alcohol strategy. Interviewees were six men and eight women with a mean age of 47 years, all white British. Specific job titles are not reported to preserve anonymity. Leadership roles included medical director, primary care network clinical director, chair of ICB prevention board, clinical lead, strategic manager, head of policy, consultant, director, manager and chair of local medical committee. Professional specialisms included GP, pharmacist, public health, population health, psychiatrist and gastroenterologist. We also interviewed third sector partners in public involvement and engagement with a focus on upstream population-wide prevention rather than downstream specialist service provision. Individual semistructured interviews were conducted by MM, a sociologist and senior qualitative researcher, via video conference using a topic guide (online supplemental file 1) between December 2022 and January 2023. Interviews lasted between 38 and 75 min. These covered the following topics:

- ICS role and how attention to alcohol use fitted within the newly evolving system, including how alcohol was (or was not) being addressed and by whom.
- Views on current primary care practice relating to alcohol, particularly in relation to polypharmacy and Core20PLUS5 approach to reducing healthcare inequalities.^14^
- Thoughts on reframing alcohol specifically as a drug as a potential leverage point for addressing alcohol in the ICS and NHS, with implications for clinical practice.^22 23^

The latter idea originated in promising findings from previous work exploring whether and how pharmacists may clinically incorporate alcohol in medication reviews, an idea also well received by patients.^19 24 25^ Audio recordings were professionally transcribed verbatim. Coding in NVivo V.1.0 by the lead author supported a constructionist thematic analysis.^26^ Data on addressing alcohol harm in the ICS was summarised and patterns of shared meaning were identified, noting common, recurring and conflicting perspectives. Preliminary analysis of sample scripts and the final analytic narrative were discussed with coinvestigators.

### Patient and public involvement

Among the stakeholders interviewed, public involvement was represented. There was no direct involvement of patients in this particular study. Coproduction of the wider research programme involved patients within the research team and other forms of patient involvement.^27^

## Results

Stakeholders reported working in stretched and underfunded circumstances, trying to map resources and develop priorities across diverse organisational units, seeking to align these with ideas forming at a system level. Systems’ approaches encourage bigger picture thinking by focusing attention on how actors, services and organisations interconnect and influence each other.^28^ Systems’ thinking on alcohol harm prevention was absent in ICSb and nascent in the one with an alcohol strategy (ICSa). The latter had long-standing recognition of the harm alcohol does regionally and hoped to link to prevention and health inequalities agendas.

### Prioritising treatment over prevention

Stakeholders consistently noted that alcohol initiatives were usually focused downstream. They provided a range of strategic and practice orientated explanations.

#### Strategic factors

Local authority commissioners were working to rebuild defunded alcohol services with ICBs and to support hospitals with the highest rates of alcohol dependence-related admissions to establish alcohol care teams. Funding for this through the LTP was welcomed by public health leaders:

… prior to that everybody was … look[ing] to public health as being the ones who should provide the services and the budgets were limited (S5).

Interviewees identified lack of progress on alcohol in the NHS as part of the bigger prevention problem. A GP who had played a key role in initiating prevention approaches for many years, and in the development of the current alcohol strategy in their ICS, likened the NHS to a ‘machine’ which was hard to adjust:

… like a very slow juggernaut … [it’s] slowly turning its direction to more preventative approaches. But … the centre is very deeply rooted in biomedical models … as a result, even when they are talking prevention, they [NHS] sometimes struggle to get beyond the things that impact on them (S11).

Key system levers or mechanisms for progress on alcohol identified by this interviewee were, “an enormous shift in what our workforce culturally think of as their role, and [are] capable of delivering, and [are] confident and enthused by,” and “whether we seriously involve our communities, and … invest in community assets and asset-based community approaches” (S11). All interviewees identified the importance of getting the workforce engaged with this broader perspective and able to contribute. For the ICB-led alcohol strategy (ICSa), this meant, “winning hearts and minds” (S1), that is, getting NHS staff to recognise and acknowledge alcohol harm as an issue and gain a sense of the implications for their own roles. This was said to be important for two reasons, first, “because they’re the army” and, second, because harmful drinking was “reflected in our workforce” (S1). Following a survey, health and social care staff across this ICS were being offered coaching to address their own drinking. A directory of alcohol services to support staff working across the ICS and a programme of alcohol studies via an e-learning platform were in development.

ICSb was less structurally developed and focusing on interventions promoted in existing national guidance. A public health team at one local authority offered free, one-off, alcohol identification and brief advice training to non-alcohol specialists and found take up to be low. The leader of this team, however, who was also working across the ICS, wanted more upstream intervention and aspired to introduce local level minimum unit pricing, but was struggling with feasibility issues: “[in terms of] the things that we know work around alcohol on a population level, you feel very helpless” (S6).

#### Practice factors

Primary care leaders in both ICSs explained that prevention orientated work on alcohol was not a practice priority given that this was not financially incentivised and because, “when there’s an overwhelming demand on the system and you’re firefighting” (S4), the focus is on the most acute and immediate problems:

This feels like a slightly distant conversation for us, [in primary care] at the minute, because [of] all the practicalities of forming an organisation [PCNs], at the same time as taking on quite a lot of pressure from elsewhere in the system …. we’ll never solve the acute hospital pressures if we don’t get on top of prevention … But it’s just too soon … we’ve tried and failed to do prevention about five or six times before and every few years it comes back around … we’ve never managed to do it properly and, in the context of the world that we’re working in just now, I’m not entirely convinced that we’ll manage to get it right this time either (S10).

Alcohol practice in the current system was seen as reflecting the wider cultural blindness to alcohol as a health harming drug:

It’s so embedded in the culture … I don’t think of it like a drug in those terms … even with diabetes, I’m thinking more about the sugar content of wine and beer, than I am the ethanol component … there definitely is a lot of scope, but I think the barriers … are not just medical … they’re cultural and social … As a society, we don’t treat alcohol like a drug (S4).

Interviewees in both ICSs recognised the disjunctions between aspirations and achievements and the current disconnects between upstream and downstream interventions. Primary care leaders said that brief interventions would need to be more valued and encouraged, form part of the GP contract and be supported by wider alcohol interventions. Local authority public health teams commissioned specialist alcohol services, with limited resources and powers to advance preferred upstream interventions. NHS priorities were focused downstream on treating alcohol dependence and its consequences in hospitals.

### Alcohol and reducing healthcare inequalities

All interviewees had heard of the NHS CORE20PLUS5 approach to reducing healthcare inequalities.^14^ Some leaders with an alcohol focus, and a clinical rather than public health background, expressed the view that although alcohol was not specifically highlighted in this, it may not matter because it was now implicit in the work of the contemporary NHS:

… people from a more deprived background suffer more harms, independent of … intake … I don’t know how you would work in the field or work in healthcare without noticing it but we certainly talk about it a lot (S2).

Interviewees in primary care had less knowledge of Core20PLUS5 and most had not thought about it in relation to alcohol:

… it’s presented to us by public health, but it’s not something we’re talking about in primary care … it’s a level detached from us … we’re being told … that’s for us as commissioners, but it’s not filtering down at a provider level yet (S9).

… we’d initially be thinking about smoking, rather than alcohol … I don’t think it [alcohol] does have the visibility … that it could and perhaps should have (S10).

One public health leader noted the persistent ‘power of tobacco’ and suggested that alcohol could have similar prominence:

… they re-released that diagram of Core20PLUS5 with smoking across all five of the clinical areas … but … you could substitute alcohol across all five of those areas … The long-term plan … is all to do with the alcohol care teams and … I haven't had any conversation so far about alcohol within [Core20PLUS5] (S6).

Similarly, another public health leader noted that, apart from treatment, alcohol was often left out of NHS strategy documents:

… the cancer plan … didn’t mention alcohol at all … how can you have a cancer plan without talking about alcohol? … (S5).

### Seeing alcohol differently, as a clinically important drug

Reframing alcohol as a clinically important drug resonated with all the stakeholders. They recognised that despite alcohol use being an important consideration for treatment effectiveness at a clinical level, it was not currently considered in these terms:

… tagging it in as part of polypharmacy and as a drug within that to be optimised … strength, dose, timing … like we would do any drug, is actually probably a very good tack to go from, from a clinician point of view (S3).

… talk[ing] about it in a scientific way [may] get … health to do something different with it, as opposed to ignore it … [and] write it off as a social [issue] (S12).

An interviewee with a public health background saw this as ‘wise’ for a primary care audience, but:

… if I were trying to talk to government ministers about alcohol, I'd be trying to make it as non-clinical as possible, because I’d be trying to persuade them that the social determinants of alcohol use are really key (S6).

This underlines the care with which alcohol needs to be discussed as posing issues for the NHS that need to be addressed. A stakeholder in public engagement noted that ‘medicalisation’ was a tactic that had been successfully used in addressing tobacco harm:

… we’ve medicalised the language around tobacco … usage, which I think has been hugely compelling in terms of getting that up the NHS agenda … away from … being a behavioural thing to … being essentially an addiction … that needs medical treatment (S14).

While framing alcohol as a drug made sense to these key stakeholders, interviewees recognised that many health professionals were not confident with the subject, and thus need support to talk to people about such a sensitive issue (S2, S5, S7, S9). This would mean moving away from template-driven lifestyle questions (S1, S8) and generic stock information giving (S9, S10). Giving more meaningful attention to alcohol carries obvious risks for clinicians of not being able to offer more as things stand:

I know full well that’s [current practice] meaningless and it has a very poor outcome, they need structured support and … monitoring … if you haven’t got the resource to make that happen as a clinician, that’s probably the bit that makes you shy away … (S9).

Shying away from talking about alcohol was largely understood as how practitioners dealt with the existing constraints of the system, because patients were very used to being asked about all sorts of potentially sensitive things. In addition to organisational priorities and capacity, this reluctance was attributed to the normalisation of alcohol use, making it potentially challenging to raise. A patient involvement lead said:

… the default setting in health at the minute is get them in, get them out, speak for as little time as possible …. making it impossible for ourselves before we even start … alcohol isn’t … embedded … [in] understanding you and the things that will impact your health, it’s completely divorced from that … (S8).

Some stakeholders identified examples in primary care where alcohol had been overlooked in their own work, for example, in producing antidepressant deprescribing guidance:

… alcohol is not mentioned anywhere in that … it should be, because … you don’t want somebody to attempt to self-medicate with alcohol, as a replacement, because, clearly, that’s not going to work and will cause all sorts of other health problems (S10).

### Barriers to change in the ICS: focus restricted to the heaviest drinkers and staff overload leading to change fatigue

Stakeholders recognised that seeing alcohol as a drug entails not just reconceptualising targets for clinical attention but also taking stock of the ways in which alcohol is relevant to the broader endeavours of improving population health by better integrating services. Various obstacles to moving in this direction were identified.

A focus only on a minority of heavy drinkers was something that leaders in public health identified as in need of change:

… we need to take the narrative away from it being a small number of people that have a problem with alcohol to … a societal problem that we need to tackle because, if we don’t, we are going to continue to see an increase in … all of these conditions, which are preventable (S5).

Greater support for heavy drinkers, however, is a need that is not going to go away. Knowledge of alcohol treatment services among primary care practitioners was thought limited and referrals sometimes problematic. One GP gave an example of “the harder cohort in the middle when you combine mental health and drinking” (S9) because psychological therapy services bounced back referrals highlighting alcohol as a problem.

Notwithstanding the appeal of the reframing, there were doubts about the practicalities of embedding changes in the current system:

… it makes absolute sense … a lot of people are damaging their health with alcohol, who aren’t dependent … How … is a challenge, especially if you’re in primary care and you’ve got eight … or ten minutes … even if you put it as a QOF, it would just get covered briefly at the end and ticked off … the mechanism of achieving that in a very burnt out and exhausted and over-demanded health service is challenging (S13).

Some interviewees articulated a general sense of fatigue from being overloaded with initiatives that sounded good but were poorly evidenced, implemented and supported, which therefore failed. Any new initiative was in danger of being perceived as telling staff that they were not doing enough and adding to already unrealistic workload expectations. Some identified a data gap across the system and a need to link alcohol to prescribing data to target advice and produce evidence. The ICSa ICB alcohol strategy was exploring how to make NHS-wide patient information available at GP practice level through useable dashboards. Interviewees had no NHS direction on alcohol outside of the LTP and public health leaders felt limited in their powers to implement evidence-informed upstream interventions. This therefore risks repeating the failures of the past in a vicious circle, with alcohol doing untold damage over time, increasing NHS workload and leaving staff, as one interviewee put it, “in survival mode, just doing the basics” (S2).

## Discussion

In the absence of any wider direction from the NHS, the ICS with an ICB-led alcohol strategy had made an innovative start with an approach focused on NHS staff. Any action on alcohol in the other ICS was without any overall strategy. Ongoing funding shortfalls, and the wider issues of embedding the logic of prevention within the NHS, meant that existing services were seen as too reactive. A narrow biomedical model focus on individual alcohol harm from heavy drinking served to externalise prevention as an issue for elsewhere in the system.

This is a modest study, with potentially major policy implications to be teased out. Purposive sampling sought to generate in-depth insights that may be helpful more widely, rather than to strive for ICS representativeness. The resulting sample, while small, provides a useful pragmatic snapshot of early ICS approaches to alcohol from the perspective of senior leaders and third sector partner key informants. The interviewees were knowledgeable, spoke articulately about system features, were open to discussing what changes are needed and considered how they may relate to existing NHS strategies. Both ICSs were in the north of England and the data collection was undertaken by a single researcher (MM) who also led the analysis. The interviewer elicited thoughtful accounts and the analysis has yielded a coherent account of major issues whose strength and limitations may be appreciated directly by the reader. Nonetheless, the transferability of the findings beyond the specific contexts of the two ICSs is uncertain. These study limitations should be borne in mind when interpreting the findings themselves, as well as their generalisability.

ICSs in England are in the early days of system development envisioned as a group of interconnected parts working together for a specific purpose.^29^ Studies show that ICSs are still developing their ‘architecture’,^30^ with a ‘short-termist’ approach from government leading to focus on immediate operational priorities over the stated long-term goals of integration, equity and prevention.^31^ Pressures on primary care have fuelled the medicalisation and pathologisation of what are inherently social and structural issues, for example, overprescription of antidepressants as a response to poverty-related distress.^32^ At the same time, the clinical consequences caused by the production, distribution and consumption of the readily available psychoactive drug alcohol (ethanol) continue to be overlooked.^19^ Health services research on alcohol in the NHS and other health systems is underdeveloped. While the role of social factors in health and health inequalities is increasingly headlined in policy, services remain focused on individual behaviour with insufficient attention paid to the means and extent to which alcohol harm is a product of economic and social forces external to and acting on the individual.^7 33^ Such issues form part of the emerging literature on the commercial determinants of health.^34^

This study identifies a need for further research on how the ICS vision is implemented in practice and a national strategy deficit on how ICSs who want to act on alcohol can be supported. Should the ICS structure be determined as the right way to proceed, given few ICSs currently prioritise alcohol, there is a need for a national strategy to encourage and support ICS action on alcohol.^7^ An emphasis on alcohol as a drug, attention to which can be integrated into the everyday clinical work of the NHS, has potential to ameliorate the policy and practice distance from prevention objectives, particularly in primary care. However, practitioners cannot effect far-reaching change within health systems in isolation. There is a scope for ICSs to be important agents of change. A national prevention strategy could determine how the NHS seeks to manage alcohol in its own work and how this links to other efforts to improve public health.

## supplementary material

10.1136/bmjph-2023-000829 online supplemental file 1

## Data Availability

No data are available.
