# Supplementary material for: Barriers and leverage points for seeing alcohol differently in integrated care systems in England: a senior stakeholder interview study
Source: BMJ Public Health. 2024 Jul 31;2(1):e000829. doi: 10.1136/bmjph-2023-000829 (PMC11812887; doi:10.1136/bmjph-2023-000829)
Supplement: online supplemental file 1 [file bmjph-2-1-s001.pdf]

Supplementary file: ICS interview guide

Intro self. Will ask you about:

- Your role in the ICS (we know it's a slow, protracted reorganisation)
- How attention to alcohol use fits in the new ICS; how it is (or is not) being addressed and by whom (Gaps. Plans. Whether the focus is on treatment or prevention initiatives or both?)
- Views on current **primary care** practice relating to alcohol, particularly alcohol in relation to polypharmacy and Core20PLUS5 key clinical areas of health inequalities.
- Views on our proposed intervention – the Medicines and Alcohol Consultation
- Thoughts on potential barriers to this and how it might be used as a potential leverage point for addressing alcohol in the system
- Thoughts on the implications of an enhanced alcohol role for the pharmacy profession

Remind recording and confidentiality, check any questions and OK to continue

Interview

1. Clarify role and ICS
    - Full job title
    - How long have you been in role… (what attracted you to it?) (role juggling?)
    - Are you full-time/part-time?
    - ICS came in formally in July, where is yours up to in its development? Any particular challenges at the moment?

2. How alcohol features in the system [as a drug?]
    - To what extent do you think alcohol is a priority in the ICS? (Overshadowed by other concerns?)
    - Is there a strategy for addressing alcohol use? As a population health issue and/or a treatment issue? Are these well-connected? Differences between place and region?
        - Long Term Plan says, '*alcohol contributes to conditions including cardiovascular disease, cancer and liver disease, harm from accidents, violence and self-harm, and puts substantial pressure on the NHS*'. The focus in the plan is on preventing alcohol dependence-related admissions to hospital – is this the focus of the ICS?
        - At population level the evidence supports restricting availability and advertising and raising prices. Any thoughts on ICS's role in relation to this [any links to economic and environment strategies]?
    - Do you know how alcohol is currently addressed in primary care and how this links to other parts of the ICS?
        - Thoughts on provision of screening, BI and treatment

- Part of our approach is to consider alcohol as a drug in the system which, in addition to the usual focus on potential dependence, widely impacts health and medication. So, not as a standalone lifestyle or healthy living issue but a clinical issue that impacts healthy life expectancy. What do you think about this?
- Is alcohol being addressed in the ICS as part of tackling health inequality e.g. via the Core20PLUS5?
    - PLUS5 mentions alcohol dependence but alcohol is also directly implicated in the key clinical areas requiring accelerated improvement to tackle health inequalities = hypertension, mental illness, cancer, COPD, maternity.

3. Your thoughts on our proposed intervention in primary care
    - To what extent are you aware of the developing Clinical Pharmacy role in PCNs? How or does your role connect with this? Knowledge of progress. Any thoughts on the benefits that may arise from the new pharmacist workforce? Any problems that might arise?
    - What about the new Structured Medication Reviews (SMRs) service that CPs are delivering in GP surgeries? [*described as a patient-centred, outcome-focused approach to medicines optimisation comprising an invited, personalised, holistic review of all medicines for people at risk of medicines-related harm, lasting 30 minutes or more to tackle problematic polypharmacy*]. Whether and how your role sits in relation to SMRs and/or tackling problematic polypharmacy.
    - Our proposed intervention is to include alcohol in SMRs as another drug in the mix rather than as a standalone 'lifestyle' issue to be addressed separately. What do you think of this?
        - Thoughts on alcohol as integral to clinical care (cf BI).
        - Impacts across the lifecourse but people not recognising its impact on their own chronic ill health or as another drug impacting on multiple medications. A blind spot in the system?
        - Do you think health professionals see alcohol as a sensitive topic they shy away from? Are there different communication styles about alcohol across the system? Barriers to person-centred communication?
    - What do you think about an enhanced alcohol role for the pharmacy profession? How might that link with broader strategic prevention initiatives. [Support required for clinical pharmacy role development?]
    - Thoughts on links to development of social prescribing in primary care [links to place and wider issues of safeguarding, loneliness, healthy ageing]
4. Closers
    - From your perspective, can you see any particular systemic barriers or opportunities in what we are proposing? How might the MAC be used as a potential leverage point for addressing alcohol?
    - [If relevant] What do you hope the clinical pharmacist role and SMRs will look like in five years time?

- How would you like to see alcohol being handled in the system in five years time?
- Any research on alcohol you would like to see happen? Important evidence gaps to be filled.
- Able to help with recruitment of CPs to study?

Finally
Anything they wish to add about integrating alcohol in the system? Anyone else you think we should talk to in your ICS/HWB?
